# Cardiovascular effects of mollic acid glucoside, a 1α-hydroxycycloartenoid saponin extractive from *Combretum molle* R Br ex G Don (Combretaceae) leaf

**Published:** 2008-06

**Authors:** John AO Ojewole

**Affiliations:** Department of Pharmacology, School of Pharmacy and Pharmacology, Faculty of Health Sciences, University of KwaZulu-Natal, Durban

## Abstract

**Summary:**

The cardiovascular effects of mollic acid glucoside (MAG), a 1α-hydroxycycloartenoid saponin extractive from *Combretum molle* R Br ex G Don (Combretaceae) leaf, have been investigated in some experimental animal paradigms. The plant extract (MAG, 5–80 μg/ml) produced concentration-dependent, significant (*p* < 0.05–0.001) negative inotropic and negative chronotropic effects on guinea pig isolated electrically driven left, and spontaneously beating right atrial muscle preparations, respectively. MAG also significantly reduced (*p* < 0.05–0.001) or abolished, in a concentration-dependent manner, the rhythmic, spontaneous contractions of portal veins isolated from healthy, normal Wistar rats.

Like acetylcholine (ACh, 10^-8^–10^-5^ M), the plant extract (5–80 μg/ml) produced concentration-related relaxations of rat isolated endothelium-containing thoracic aortic rings pre-contracted with noradrenaline (NA, 10^-10^–10^-5^ M). The vasorelaxant effects of MAG in the aortic rings were markedly inhibited or annulled by N^G^-nitro-L-arginine methyl ester (L-NAME, 10^-5^ M), a nitric oxide synthase inhibitor. Furthermore, MAG (2.5–40 mg/kg iv) caused dose-related, transient but significant reductions (*p* < 0.05–0.001) in the systemic arterial blood pressures and heart rates of anaesthetised normotensive and hypertensive rats.

The results of this laboratory animal study indicate that MAG caused bradycardia, vasorelaxation and hypotension in the mammalian experimental models used. The vasorelaxant action of MAG was endothelium dependent, and was therefore possibly dependent on the synthesis and release of nitric oxide (NO). The findings of this study suggest that *Combretum molle* leaf may be used as a natural supplementary remedy in essential hypertension and in certain cases of cardiac dysfunctions in rural African communities.

## Summary

The Combretaceae family of plants consists of 18 known genera, the largest of which are *Combretum* with about 370 species, and *Terminalia* with about 200 species.^1^,^2^ The Combretaceae family is well represented in southern Africa, particularly in the bushveld. More than 50 species of the family reach tree size.^3^ Plant species from the genus *Combretum*, and to a lesser extent, *Terminalia*, are most widely used for medicinal purposes in African folk medicines.^2^ The genus *Combretum*, the largest in the family Combretaceae, is represented in southern Africa by about 30 species of trees or shrubs, some of which feature prominently in traditional African medicine.^4^ *Combretum* species are also widely distributed in many other parts of Africa where they often constitute the most abundant species.^5^ Many triterpenoids and their glycosides have been isolated from the leaves of South African *Combretum* species.^5^-^8^

*Combretum molle* (R Br ex G Don) Engl & Diels (Combretaceae) is a small to medium-sized perennial, erect, terrestrial, semi-deciduous tree of about 6–10 m in height, with spreading crown. Bark of the branches exfoliates in irregular, untidy, fibrous strips or threads.^3^ The leaves, which are usually crowded near the branch tips, are narrowly elliptic or ovate-elliptic to almost circular, normally about 60–100 mm long and 40–60 mm wide, dense with velvety hairs, particularly below, with tapering apex, rounded to shallowly lobed base, and net-veining conspicuously raised below.^3^ The greenish-yellow flowers are in axillary spikes of up to 90 mm long, often appearing before or with the new leaves.^3^ The four-winged fruits are usually 15–20 mm long and 15–20 mm wide, yellowish-green and flushed with red, drying to a golden, reddish-brown colour.^3^ The wood is yellowish-brown, hard and termite resistant, and is usually used for household utensils, hut-building, and so on.^3^

Various morphological parts of *Combretum molle*, particularly the leaves and roots, are traditionally used in South African folk medicine for a variety of human ailments.^3^ Fresh or dry leaves are used for treating fevers and microbial infections, as dressings for wounds, and as snakebite antidote. Decoctions of the root bark are also used for constipation and other gastro-intestinal disorders, and to treat abortion.^9^,^10^

Pegel and Rogers^4^ have reviewed the triterpenoids extracted from the leaves of southern African *Combretum* species. According to Pegel and Rogers,^6^ the major constituent of the acetone extract of *Combretum molle* leaves is a colourless, sparingly water-soluble, crystalline triterpene acid saponin which has been named mollic acid glucoside. In a more recent study, Rogers and Thevan^8^ have shown that mollic acid glucoside is a mixture of mollic β-D-xyloside and α-L-arabinoside in an approximate ratio of 2:1. However, titration of mollic acid glucoside with sodium hydroxide (NaOH) showed that it has only one carboxyl functional group, and that it forms a highly water-soluble sodium salt.^6^ Triterpenoids, mollic acid 1α-hydroxycycloartenoid, mollic acid glucosides and their xyloside and arabinose have been isolated from the leaves of *Combretum molle* and certain other species of *Combretum*.^4^,^7^,^8^,^11^,^12^

Traditional health practitioners in many parts of Africa usually use the leaves and bark of *Combretum* species as remedies for a variety of human ailments, including hypertension, some cardiac disorders, abdominal discomfort, body pains, respiratory disorders, colds and fevers, ear and eye ailments, schistosomiasis, hookworms, dysmenorrhoea and infertility in women, leprosy, syphilis, microbial infections, general body weakness, and so on.^9^,^10^,^13^ Some traditional health practitioners in KwaZulu-Natal have also claimed that decoctions, infusions and other extractives of *Combretum molle* and certain other species of *Combretum* are effective remedies for the management, control and/or treatment of an array of human ailments, including essential hypertension, cardiac dysrhythmias, and painful, arthritic and other inflammatory conditions. To date, however, reports on the pharmacological actions of *Combretum molle* extracts are sparse in the biomedical literature.

The present study was therefore undertaken to examine the cardiovascular properties of mollic acid glucoside, a 1α-hydroxycycloartenoid extractive from *Combretum molle* leaves, in experimental animal paradigms, with a view to providing a pharmacological justification (or otherwise) for some of the folkloric, ethnomedical uses of the leaf in the management, control and/or treatment of hypertension and certain cardiac disorders in some rural communities of southern Africa.

## Materials and methods

The protocol and procedures used in this study were approved by the Ethics Committee of the University of KwaZulu-Natal, and conform to the *Guide to the Care and Use of Animals in Research and Teaching*.^14^

The mollic acid glucoside powder used in this study was kindly supplied by Prof CB Rogers, who extracted, characterised and identified the plant material from *Combretum molle* R Br ex G Don (Combretaceae) leaves.

Detailed phytochemical processes leading to the isolation and characterisation of the mollic acid glucoside (MAG) residue used have been described earlier.^4^,^6^-^8^ Aliquot portions of the extract residue were weighed and dissolved in warm distilled water (at 45°C, containing one drop of normal sodium bicarbonate solution) for use on each day of our experiment.

Healthy male Dunkin-Hartley guinea pigs (*Cavia porcellus*) weighing 300–450 g, and healthy young adult male Wistar rats (*Rattus norvegicus*) weighing 250–300 g were used. The animals were kept under laboratory conditions of temperature, humidity and light and were allowed free access to food (standard pellet diet) and water *ad libitum*. All the animals were fasted for 16 hours but allowed free access to water before the commencement of our experiments.

Guinea pig isolated atrial muscles were used for the *in vitro* evaluation of the effects of the plant extract on myocardial contractility, while rat isolated portal veins and thoracic aortic rings were used to examine the vasorelaxant effects of the plant extract. Normotensive (normal) Wistar and hypertensive Dahl salt-sensitive rats were used for the *in vivo* investigation of the antihypertensive effect of the leaf extract (MAG).

## Guinea pig isolated atrial muscle strips

The guinea pigs were sacrificed by stunning and exsanguination. The left and right atrial muscles of the animals were isolated and mounted as previously described by Ojewole.^15^

The isolated left atrium of each guinea pig was impaled on a thin platinum wire electrode and suspended under an applied resting tension of 1.0 g in a 30-ml Ugo Basile organ bath containing Krebs-Henseleit physiological solution (of composition, in mmol/l: NaCl, 118; KCl, 4.7; NaH_2_PO_4_, 1.28; NaHCO_3_, 25.0; MgCl_2_, 1.2; CaCl_2_, 2.52; and glucose, 5.55 – pH adjusted to 7.4) maintained at 34 ± 1°C and continuously aerated with carbogen (95% O_2_ + 5% CO_2_ gas mixture). Each left atrial muscle preparation was electrically driven with square wave pulses of 5-msec duration at a frequency of 3 Hz and a supramaximal voltage of 5–10 volts, delivered by an SRI stimulator.

The spontaneously beating right atrium of each animal was also set up under the same physiological experimental conditions and allowed to beat spontaneously. Two isolated electrically driven left atrial muscle strips and two isolated spontaneously beating right atrial muscle preparations were always set up at a time (one used as the test, and the other as the control preparation) to allow for changes in the atrial muscle sensitivity. The atrial muscle preparations were left to equilibrate for 45–60 minutes (during which time the bathing physiological solution was changed every 15 minutes) before they were challenged with MAG or any of the reference drugs used. The test atrial muscle preparations were treated with sequentially applied graded concentrations of MAG and/or the reference agonist drugs, whereas the control atrial muscle strips were treated with volumes of distilled water (0.1–0.6 ml) equivalent to the volumes of bath-applied MAG solution used.

In separate sets of experiments, the inotropic and chronotropic effects of MAG (5–80 μg/ml) on the atrial muscle preparations were investigated in the presence of atropine (ATR, 10^-6^ M). The electrically provoked and spontaneous contractions of the atrial muscles, as well as the MAG- and reference agonist drug-induced responses of the atrial muscle preparations were recorded isometrically by means of Ugo Basile force-displacement transducers and pen-writing Gemini recorders (model 7070).

## Rat isolated portal veins

The healthy male rats used were sacrificed by stunning and exsanguination. The abdomen of each rat was quickly opened by midline incision and the intestines were pulled aside. The portal vein of each rat, with an *in situ* length of approximately 2 cm, was carefully cleaned free of extraneous connective and fatty tissues, and then removed from the animal. Each isolated portal vein was suspended under an applied resting tension of 0.5 g in a 30-ml Ugo Basile organ bath containing Krebs-Henseleit physiological solution.

Two isolated venous tissue preparations (one control and the other MAG- or reference drug-treated test) were always set up in order to make allowance for changes in the venous tissue sensitivity. Control venous muscle strips were treated with distilled water only (ie, the vehicle in which MAG and reference drugs were dissolved). The venous tissue preparations were allowed to equilibrate for 45–60 minutes (during which time the bathing physiological solution was changed every 15 minutes) before they were challenged with MAG or any of the reference drugs used. The plant extract- (MAG, 5–80 μg/ml) and reference drug-induced responses of the venous smooth muscle preparations were recorded isometrically by means of Ugo Basile force-displacement transducers and pen-writing Gemini recorders (model 7070).

## Rat isolated thoracic aorta rings

Healthy male rats (250–300 g) were sacrificed by decapitation. The descending thoracic aorta of each rat was quickly and carefully excised and placed in a Petri dish filled with ice-cold Krebs-Henseleit physiological solution. The aorta was cleaned free of fat and excess connective tissue and cut into rings of approximately 3–4 mm in width. All dissecting procedures were carefully done to protect the functional endothelium from damage. In some aortic rings, the endothelial layer was mechanically removed by gently rubbing the luminal surface with distilled water-moistened cotton wool, followed by back and forth rubbing with a small, plastic tube.

A pair of rat isolated aortic rings, one with intact functional endothelium, and the other one with endothelium denuded, were always set up in parallel for appropriate comparison. Each of the isolated aortic rings was separately suspended under an applied resting tension of 1.0 g in a 30-ml Ugo Basile organ bath containing Krebs-Henseleit physiological solution maintained at 36 ± 1°C and continuously aerated with carbogen. The aortic tissue preparations were left to equilibrate for 45–60 minutes (during which time the bathing physiological solution was changed every 15 minutes) before they were challenged with graded concentrations of MAG or any of the reference drugs used.

At the end of the equilibration period, the aortic ring preparations were initially contracted with bath-applied noradrenaline (NA, 10^-5^ M). Endothelial integrity and successful removal of the functional endothelium was assessed by the presence or absence, respectively, of relaxant response to acetylcholoine (ACh, 10^-5^ M). Acetylcholine-induced relaxation ≤ 5% was taken as satisfactory removal of the functional endothelial layer. Such endothelium-denuded aortic muscle preparations were used in this study.

After subsequent wash out and equilibration period of 30 minutes, cumulative dose-response curves were obtained with noradrenaline (10^-10^–10^-5^ M) in the normotensive rat aortic rings with and without endothelium. Subsequently, 20 minutes’ pre-treatment of the aortic muscle preparations with graded concentrations of the plant extract (5–80 μg/ml) was carried out before the next cumulative additions of noradrenaline (10^-10^–10^-5^ M) to the bath fluid. After the addition of each NA concentration, a plateau response was obtained before the addition of the next higher dose in all cases of cumulatively applied noradrenaline concentrations. Consecutive dose-response curves were taken at 30-minute intervals, during which time the bathing Krebs-Henseleit physiological solution was changed three to five times until the tension developed returned to basal levels.

Following 20 minutes’ incubation of the aortic ring preparations with MAG (5–80 μg/ml), the arterial relaxant effect of the plant extract was examined on the endothelium-containing and endothelium-denuded aortic ring preparations pre-contracted with sequentially applied or cumulatively administered noradrenaline (10^-10^–10^-5^ M). The effect of the vehicle (distilled water) in which MAG and the reference drugs were dissolved was also tested. After each challenge, the aortic rings were washed three to five times with fresh Krebs-Henseleit physiological solution and allowed to equilibrate for 30 minutes before they were challenged again with any of the reference drugs or MAG. The contractile and/or relaxant effects of all the reference drugs used, as well as MAG-induced relaxations of the isolated aortic ring preparations were recorded isometrically by means of Ugo Basile force-displacement transducers and pen-writing Gemini recorders (model 7070).

## Systemic arterial blood pressures and heart rates of anaesthetised rats

Young adult male normotensive Wistar, and young adult hypertensive Dahl salt-sensitive rats weighing 250–300 g were used. Before the commencement of our experiments, the salt-sensitive rats were placed on 4% saline water and normal food (standard pellet diet) for six to eight weeks (during which time the arterial blood pressures of the animals rose to between 170/130 and 190/140 mmHg). Salt-sensitive rats with arterial blood pressures ≥ 170/120 mmHg were considered to be hypertensive and used in this study.

Each of the normotensive and hypertensive rats was anaesthetised with an intraperitoneal injection of 0.11 g/kg of Trapanal® [sodium 5-ethyl-(1-methylbutyl)-2-thiobarbiturate]. The right femoral vein of each rat was cannulated with a small polythene cannula for the administration of the plant extract and reference drugs. In order to minimise coagulation, heparin (500 units/kg) was intravenously administered to the animal and flushed in with 0.2 ml of 0.9% w/v sodium chloride solution. The left carotid artery of each rat was also cannulated with a small polythene cannula and connected to a four-channel Grass polygraph for systemic arterial blood pressure recording. The trachea of each rat was cannulated for artificial respiration, but the animal was allowed to breathe spontaneously. The rat’s body temperature was maintained at 36 ± 1°C with an incandescent lamp placed over the animal’s abdomen.

After 20 minutes’ stabilisation period, systemic arterial blood pressure (systolic, diastolic and mean arterial pressures) and heart rate of each rat were measured and recorded. The effects of MAG (2.5–40 mg/kg) and the reference drugs [acetylcholine (0.5–4.0 μg/kg iv) and noradrenaline (0.5–4.0 μg/kg iv)] on systemic arterial blood pressures and heart rates (calculated from the ECG limb lead II recording at a fast paper speed of 25 mm/sec) were recorded by means of a four-channel Grass polygraph recorder (model 79D).

In some of the rats, the hypotensive (depressor) effect of the plant extract (2.5–40 mg/kg iv) was examined after atropinisation [pre-treatment of the rats with atropine sulphate (1.5 mg/kg ip) 18–24 hours before use]. Because MAG and other drugs used in this study were dissolved in distilled water, rats treated with distilled water (2 ml/kg iv) alone were used as control animals under the same experimental conditions.

## Compounds and drugs used

The following compounds and drugs were used: mollic acid glucoside (MAG), a 1α-hydroxycycloartenoid saponin extractive from *Combretum molle* R Br ex G Don (Combretaceae) leaf; acetylcholine chloride (Sigma, England); (-)-noradrenaline hydrochloride (Sigma, England); atropine sulphate (Sigma, England); N^G^-nitro-L-arginine methyl ester (L-NAME) (Sigma, England); Trapanal® [sodium 5-ethyl-(1-methylbutyl)-2-thiobarbiturate] (Byk Gulden, Konstanz, Germany); (±)-propranolol hydrochloride (Sigma, England); calcium chloride and potassium chloride (Sigma, England). The drugs were dissolved in distilled water each day at the beginning of our experiments. Drug concentrations and doses quoted in the text refer to the salts, except the plant extract, and denote final organ bath concentrations in the *in vitro* experiments.

## Data analysis

Data obtained from test guinea pig isolated atria, rat isolated portal vein, rat aortic ring strips, and anaesthetised normotensive and hypertensive rats treated with MAG alone, as well as those obtained from distilled water-treated control isolated atria, portal veins, aortic rings and anaesthetised rats, were pooled and expressed as means (± SEM). Statistical comparison of the differences between the plant extract- and reference drug-treated test means, and distilled water-treated control means, was performed with GraphPad InStat software (version 3.00, GraphPad Software, San Diego, California, USA) using one-way analysis of variance (ANOVA; 95% confidence interval), followed by the Tukey-Kramer multiple comparison test. Values of *p* ≤ 0.05 were taken to imply statistical significance.

## Results

## Guinea pig isolated atrial muscle preparations

**Fig. 1.**
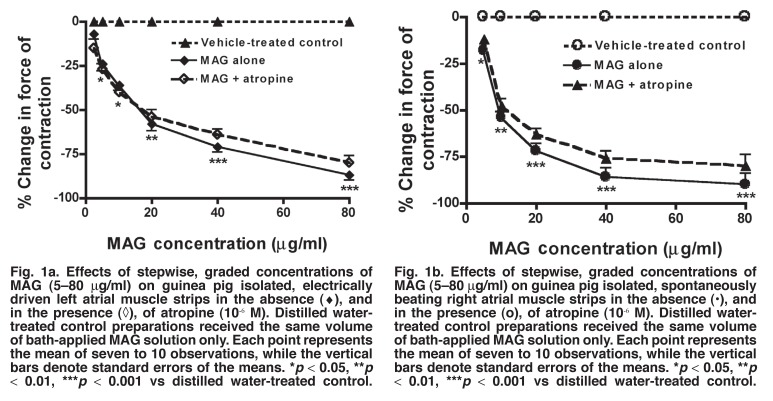
Fig. 1a. Effects of stepwise, graded concentrations of MAG (5–80 μg/ml) on guinea pig isolated, electrically driven left atrial muscle strips in the absence (♦), and n the presence (◊), of atropine (10^-6^ M). Distilled water-treated control preparations received the same volume of bath-applied MAG solution only. Each point represents the mean of seven to 10 observations, while the vertical bars denote standard errors of the means. *p < 0.05, **p < 0.01, ***p < 0.001 vs distilled water-treated control. Effects of stepwise, graded concentrations of MAG (5–80 μg/ml) on guinea pig isolated, spontaneously beating right atrial muscle strips in the absence (•), and in the presence (o), of atropine (10^-6^ M). Distilled water-treated control preparations received the same volume of bath-applied MAG solution only. Each point represents the mean of seven to 10 observations, while the vertical bars denote standard errors of the means. *p < 0.05, **p < 0.01, ***p < 0.001 vs distilled water-treated control.

Sequential administrations to the bath fluid of low to high concentrations of MAG (5–80 μg/ml) significantly reduced (*p* < 0.05–0.001) or abolished the force of contractions of guinea pig isolated electrically driven left atrial muscle preparations in a concentration-related manner (Fig. 1a). The negative inotropic effect of MAG on electrically driven left atrial muscle strips of guinea pigs was not significantly altered (*p* > 0.05) by prior exogenous administration of atropine (ATR, 10^-6^ M) to the bath fluid (Fig. 1a). The plant extract also significantly reduced (*p* < 0.05–0.001) or abolished the rate of contractions of guinea pig isolated spontaneously beating right atrial muscle preparations in a concentration-dependent manner (Fig. 1b). However, the negative chronotropic effect of MAG on the right atrial muscle strips was not significantly modified (*p* > 0.05) by prior exogenous administration to the bath fluid of atropine (10^-6^ M) (Fig. 1b), which reduced or abolished the negative chronotropic effect of acetylcholine (7.5 3 10^-8^–3.5 3 10^-6^ M) on six other spontaneously beating right atrial muscle preparations examined (data not shown).

The plant extract significantly reduced (*p* < 0.05–0.001) or abolished, like propranolol (10-9–10-5 M), the positive inotropic and chronotropic effects of noradrenaline (10^-10^–10^-5^ M) on all the other eight isolated atrial muscle strips tested (data not shown). The plant extract also significantly inhibited (*p* < 0.05–0.001) or abolished calcium-induced (Ca^2+^, 5–40 mM) positive inotropic and chronotropic responses on all the other nine atrial muscle strips examined (data not shown).

## Rat isolated portal veins

**Fig. 2.**
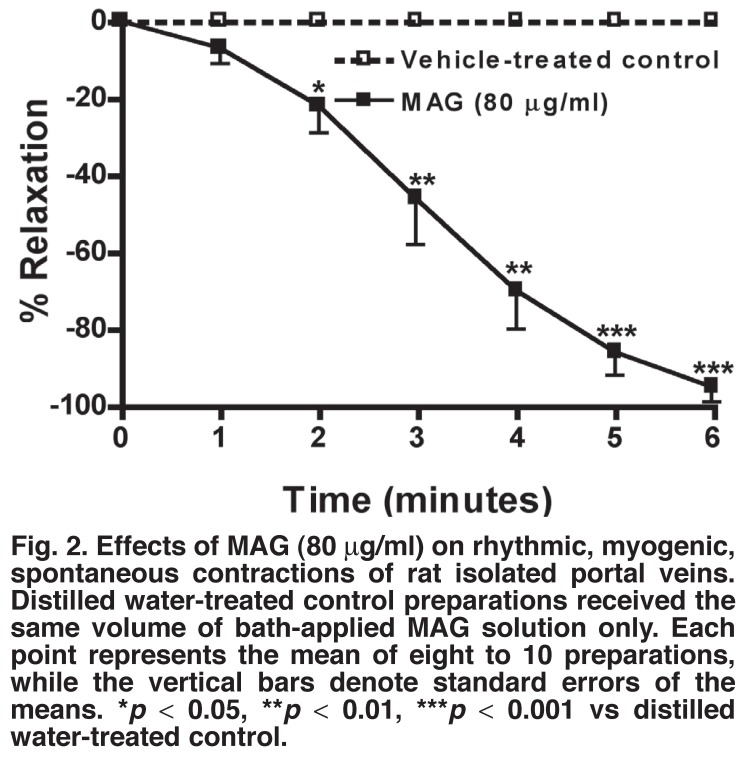
Effects of MAG (80 μg/ml) on rhythmic, myogenic, spontaneous contractions of rat isolated portal veins. Distilled water-treated control preparations received the same volume of bath-applied MAG solution only. Each point represents the mean of eight to 10 preparations, while the vertical bars denote standard errors of the means. *p < 0.05, **p < 0.01, ***p < 0.001 vs distilled water-treated control.

Sequential administrations to the bath fluid of low to high concentrations of MAG (5–80 μg/ml) always induced concentration-dependent relaxant (inhibitory) effects on the amplitude and frequency of the rhythmic myogenic contractions of the rat portal veins (Fig. 2). At the same concentration range, the plant extract also inhibited or abolished in a concentration-dependent manner contractions of the venous muscle preparations induced by noradrenaline (10-10–10-5 M) or potassium (K+, 5–40 mM).

## Rat isolated aortic ring strips

Cumulative additions of stepwise, graded concentrations of noradrenaline (10^-10^–10^-5^ M) to the bath fluid provoked concentration-dependent contractions of both endothelium-containing and endothelium-denuded normotensive rat isolated aortic ring strips, with a maximum of 3.76 ± 0.30 g tension developed. Acetylcholine (10^-8^–10^-5^ M) provoked concentration-related significant relaxations (*p* < 0.05–0.001) of endothelium-containing aortic ring preparations pre-contracted with bath-applied noradrenaline, but did not significantly relax (*p* > 0.05) endothelium-denuded aortic ring preparations pre-contracted with bath-applied noradrenaline.

**Fig. 3.**
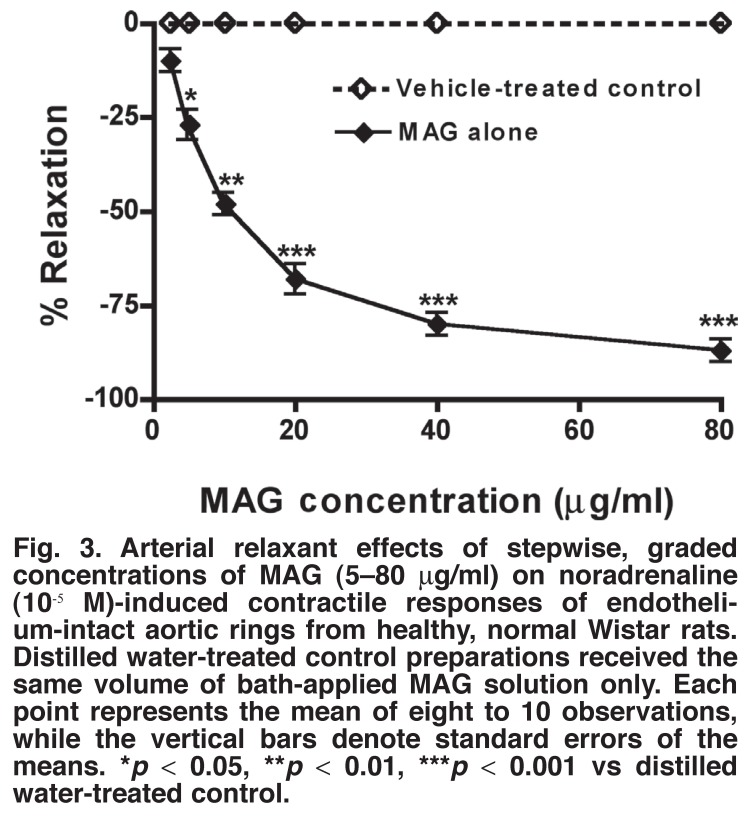
Arterial relaxant effects of stepwise, graded concentrations of MAG (5–80 μg/ml) on noradrenaline (10^-5^ M)-induced contractile responses of endothelium-intact aortic rings from healthy, normal Wistar rats. Distilled water-treated control preparations received the same volume of bath-applied MAG solution only. Each point represents the mean of eight to 10 observations, while the vertical bars denote standard errors of the means. *p < 0.05, **p < 0.01, ***p < 0.001 vs distilled water-treated control.

Like acetylcholine, sequential administrations to the bath fluid, of low to high concentrations of MAG (5–80 μg/ml) produced concentration-dependent significant relaxations (*p* < 0.05–0.001) of the endothelium-containing, normotensive rat aortic ring preparations pre-contracted with noradrenaline (Fig. 3). However, the plant extract did not significantly relax (*p* > 0.05) endothelium-denuded aortic ring preparations pre-contracted with bath-applied noradrenaline. Moreover, the plant extract shifted cumulatively administered noradrenaline concentration-response curves to the right in a non-parallel and non-competitive fashion, and suppressed NA-induced maximal contractions of endothelium-containing aortic ring muscle preparations.

Ten minutes’ prior incubation of the endothelium-intact aortic ring tissues with N^G^-nitro-L-arginine methyl ester, a nitric oxide synthase inhibitor (L-NAME, 10^-5^ M), inhibited or abolished MAG- (or ACh)-induced relaxations of the endothelium-containing aortic rings pre-contracted with noradrenaline. Ten minutes’ prior incubation of the aortic ring tissues with atropine sulphate (10^-6^ M) also inhibited or abolished acetylcholine- but not MAG-induced relaxations of the endothelium-containing aortic ring preparations pre-contracted with noradrenaline.

## Systemic arterial blood pressures and heart rates of normotensive and hypertensive rats

Acute intravenous administrations of low to high doses of MAG (2.5–40 mg/kg iv) into anaesthetised normotensive and hypertensive rats always produced transient, dose-related significant reductions (*p* < 0.05–0.001) in the systemic arterial blood pressures and heart rates of these rats (Tables 1, 2). The transient hypotensive (antihypertensive) effect of the plant extract persisted for 10–50 minutes, depending on the MAG dose administered. Furthermore, the plant extract dose-dependently inhibited or abolished the pressor effects of noradrenaline (0.5–4.0 μg/kg iv) on systemic arterial blood pressures and heart rates of the animals.

**Table 1:** Effects Of MAG (2.5–40 MG/KG IV) On Systemic Arterial Blood Pressures And Heart Rates Of Healthy Normotensive Rats. Each Va Lue Is The Mean (± SEM) Of Observa Tions From Eight Rats

		*After treatment: MAG dose (mg/kg iv)*				
*Cardiovascular parameter*	*Before treatment: control values*	*2.5*	*5*	*10*	*20*	*40*
Systolic BP (mmHg)	125.6 ± 4.2	114.4 ± 4.7	93.5 ± 4.4*	76.5 ± 4.3**	61.4 ± 4.2***	44.4 ± 3.6***
Mean BP (mmHg)	110.5 ± 4.3	100.6 ± 4.4	86.5 ± 4.5*	69.6 ± 4.2**	54.4 ± 4.5***	40.4 ± 3.3***
Diastolic (mmHg)	96.4 ± 4.6	81.4 ± 4.8	66.4 ± 4.1*	50.4 ± 4.6**	36.3 ± 4.9***	21.3 ± 3.1***
Heart rate (beats/min)	348.7 ± 18.6	321.6 ± 18.2	302.4 ± 16.7*	283.6 ± 14.4**	240.4 ± 12.2***	218.5 ± 10.4***

*p < 0.05; **p < 0.01; ***p < 0.001 vs control.

**Table 2:** Effects Of MAG (2.5–40 MG/KG IV) On Systemic Arterial Blood Pressures And Heart Rates Of Hypertensive Rats. Each Value Represents The Mean (± SEM) Of Observa Tions From Eight Rats

		*After treatment: MAG dose (mg/kg iv)*				
*Cardiovascular parameter*	*Before treatment: control values*	*2.5*	*5*	*10*	*20*	*40*
Systolic BP (mmHg)	183.6 ± 7.0	171.6 ± 6.4	158.5 ± 6.1*	143.4 ± 6.3**	126.4 ± 4.4***	104.4 ± 4.1***
Mean BP (mmHg)	144.6 ± 6.8	130.6 ± 6.1	116.4 ± 5.6*	102.4 ± 5.6**	89.5 ± 4.4***	72.4 ± 4.4***
Diastolic (mmHg)	122.5 ± 6.4	106.4 ± 4.8	91.4 ± 5.3*	74.4 ± 4.0**	60.4 ± 4.5***	44.3 ± 4.0***
Heart rate (beats/min)	392.8 ± 20.5	380.6 ± 18.4	356.6 ± 16.4*	325.4 ± 15.3**	296.6 ± 14.0***	246.4 ± 12.4***

*p < 0.05; **p < 0.01; ***p < 0.001 vs control.

Pre-treatment of the anaesthetised normotensive and hypertensive rats with atropine sulphate (1.5 mg/kg ip, 18–24 hours before use) abolished or markedly reduced the depressor effects of acetylcholine (0.5–4.0 μg/kg iv) on systemic arterial blood pressures and heart rates of the animals. However, the depressor effects of MAG on systemic arterial blood pressures and heart rates of the rats were not affected by pre-treatment of the animals with atropine sulphate.

## Discussion

The results of this study indicate that mollic acid glucoside, a 1α-hydroxycycloartenoid saponin extractive from Combretum molle leaf, possess cardiodepressant, vasorelaxant and hypotensive (antihypertensive) effects in the experimental animal paradigms used.

Furchgott and Zawadzki^16^ first described the involvement of the endothelium-derived relaxing factor (EDRF), which was subsequently determined to be nitric oxide (NO) or NO derivatives synthesised from guanidine groups of L-arginine.^17^,^18^ Endothelium-dependent relaxation, which has been demonstrated in many vascular preparations, including some veins, arteries and microvascular vessels, occurs in response to stimulation by a variety of substances, such as acetylcholine, adenine nucleotides,^19^ thrombin, substance P,^20^ calcium ionophore A23187, bradykinin and histamine.^21^ The vasodilatory effects of endothelium-dependent substances can be inhibited by several L-arginine analogues, such as N-monomethyl-Larginine (L-NMMA) and NG-nitro-L-arginine methyl ester (LNAME)^17^,^22^-^24^

Endothelial nitric oxide plays a vital role in the control of vasomotor tone and structure.^19^,^25^ On the other hand, vascular tone plays an important role in the regulation of arterial blood pressure. The development and maintenance of hypertension has been suggested to involve a reduced endothelium-dependent vasodilator influence on the vascular tissue.^25^ Impairment of endothelium-dependent vascular relaxation in human and experimental hypertension has been observed by Luscher and Vanhoutte,^19^ and the ability of nitric oxide to maintain vascular tone has been shown to be deficient in this condition.^25^,^26^ Because NO is a potent vasodilator, a deficient production and/or release of endothelium-derived NO will result in diminished vasodilator tone, thus allowing vascular resistance to rise, and this, in turn, will lead to elevated blood pressure.^19^,^25^

Relaxation of vascular smooth muscle by NO involves a series of steps. Nitric oxide is formed in functional endothelium by the activation of nitric oxide synthase (NOS), which uses L-arginine as a substrate. Once formed, NO diffuses out of the endothelium, with some entering the underlying vascular smooth muscle where it binds to and activates soluble guanylate cyclase.^25^ This enzyme catalyses the conversion of guanidine triphosphate (GTP) to cyclic guanidine monphosphate (cGMP), which in turn, causes relaxation of the vascular smooth muscle cells.^17^,^25^,^27^,^28^

In pathological conditions of the cardiovascular system, there is a dysfunction in the integrity of the vascular endothelium with a subsequent reduction in the release, bioavailability and/or action of nitric oxide.^25^ NO release and function have been shown to decrease in cardiovascular diseases such as hypertension, 19 atherosclerosis^29^ and congestive heart failure.^30^ Therefore, the development of vasodilators which can restore the level and integrity of NO in the vascular system would potentially contribute to the treatment of these cardiovascular diseases.^25^

In the present study, MAG, like acetylcholine, caused concentration-dependent relaxations of the normotensive rat isolated endothelium-containing aortic ring preparations pre-contracted with noradrenaline. This vasorelaxant property of MAG would appear to contribute, at least in part, to the antihypertensive (hypotensive) effect of the plant extract. The arterial muscle relaxant effect of the plant extract disappeared on removal of the functional endothelium. Furthermore, pre-treatment of the endothelium-containing aortic ring preparations with L-NAME, a nitric oxide synthase inhibitor, inhibited or abolished the vasorelaxant effect of MAG.

Taken together, these observations would appear to suggest that the vasorelaxant effect of MAG, like that of acetylcholine, was dependent on the formation and/or synthesis and release of endothelium-derived nitric oxide, since removal of the functional endothelial cells led to absence of relaxant response to the plant extract in the endothelium-denuded aortic ring preparations. These observations are in agreement with the findings of Baisch *et al.*,^17^ Yin *et al.*,^25^ Ignarro *et al.*,^31^ Kang *et al.*,^32^-^34^ and Martin *et al.*^35^

The present study also suggests that the endothelium-dependent vasorelaxant effect of MAG could be mediated via endothelial NO signaling in the aortic tissue preparations. However, the release of endothelial NO and the opening of potassium channels have also been implicated in the vasorelaxant effects of extracts from some other medicinal plant extracts.^36^-^38^

Noradrenaline-induced contractions of blood vessels have been shown to be partly due to calcium release from intracellular storage sites and partly due to the influx of extracellular calcium into the cell via receptor gated-channels following alpha_1_- (α_1_-) adrenoceptor activation.^39^ In the present study, endothelium-containing aortic rings pre-contracted with NA in the bathing solution with (and without) normal calcium concentrations were relaxed by exogenous additions of MAG or acetylcholine. Moreover, the non-parallel shift of the noradrenaline concentration-response curves to the right by the plant extract seems to suggest a mechanism of non-competitive α_1_-adrenoceptor blockade. This hypothesis is consistent with the work of Abreu *et al.*^40^ on ethanolic extract of *Jatropha gossypiifolia* Linn in rats.

The findings of the present study show that MAG induced vasorelaxation in healthy normotensive rat isolated portal veins and endothelium-containing aortic rings; and caused hypotension in anaesthetised normotensive and hypertensive rats. Although α1-adrenoceptor blockade may partially have contributed to the hypotensive effect of the plant extract, the experimental evidence obtained in the present study tends to suggest that vasorelaxation might largely be responsible for the hypotensive action of the plant extract. This vasorelaxant effect of the plant extract was probably mediated through endothelium-dependent NO production and cGMP release, and not related to activation of vascular endothelial muscarinic receptors. Although the precise mechanism of the hypotensive action of MAG could not be established in the present study, we excluded involvement of cholinergic mechanisms.

In conclusion, the findings of the present study lend pharmacological credence to the suggested folkloric, ethnomedical uses of Combretum molle leaves as a natural supplementary remedy in the management, control and/or treatment of hypertension and certain cardiac disorders in some rural African communities.
